# Not on the right rung for me? How status inconsistency leads to avoidance of status-threatening ties in NCAA

**DOI:** 10.1371/journal.pone.0308352

**Published:** 2024-09-23

**Authors:** Keehyuk Ra, Bo Kyung Kim

**Affiliations:** 1 Management & Organization, Foster School of Business, University of Washington, Seattle, Washington, United States of America; 2 Management, Yonsei School of Business, Yonsei University, Seoul, Korea; KAIST: Korea Advanced Institute of Science and Technology, REPUBLIC OF KOREA

## Abstract

This study examines the impact of status inconsistency on status-threatening activities within NCAA Division I men’s basketball teams. Specifically, we focus on a nested form of status that includes both individual and group-level elements. We argue that organizations dealing with status inconsistency stemming from such nested form face challenges in reducing status inconsistency. To maintain their deserved status, these status-inconsistent organizations tend to avoid activities that could further threaten their status, despite potential economic gains. An analysis of NCAA Division I men’s basketball scheduling data from 2000 to 2019 provides robust support to our theoretical arguments. Our findings suggest that the status inconsistency between a team’s status and its conference status diminished the likelihood of scheduling games with non-Division I teams, a behavior considered counter-normative in this context. This effect is most prominent among teams in “Mid Major” conferences, while teams with recent participation in the NCAA Tournament show a mitigated effect.

## Introduction

Organizational status, defined as an organization’s relative position in a status hierarchy [1–4], has significant impacts on organizational behaviors and offers various benefits to those in top positions, driving many organizations to strive for higher status [5]. While much of the status literature focuses on how status functions within a single hierarchy [6, 7], organizations often participate in multiple status systems, such as diversified firms in various industries, leading to distinct positions across these systems [8]. This status inconsistency stemming from different positions across multiple hierarchies can have crucial effects on organizational performance and behaviors [9–11]. Research on status inconsistency primarily argues that it negatively influences performance, independent of the main effect of status, and examines how organizations attempt to resolve status inconsistency issues [12, 13]. However, organizations are often nested within higher-level groups that influence their own status [14, 15], creating a unique condition for addressing status inconsistency. By examining the status difference between basketball teams and their conferences in NCAA Division I universities, this paper addresses the important question of how organizations respond to status inconsistency stemming from nested status positions.

The nested form of status inconsistency is defined as the status difference between an organization and the group to which the organization belongs [14, 16]. Unlike the other forms of status inconsistency, nested form of status inconsistency is more likely to constrain the organization’s attempt to resolve the status inconsistency due to the organization’s limited control over its group’s status, which is also composed of the status positions of all other organizations in the group. Based on this definition, we argue that status inconsistency triggers status anxiety [17, 18] because the organization feels pressure to justify the status position it deserves. Status inconsistency stemming from nested status positions, therefore, leads to avoidance of status-threatening activities. We further argue that this effect of status inconsistency is contingent upon belonging group status; status inconsistency has the strongest effect for the organizations in the middle-status group because of the uncertain nature of their social position [19]. Moreover, recent high performance weakens this effect of status inconsistency by reducing the audiences’ confusion about status evaluation and making the organization less status anxious.

We test our theoretical arguments in the context of NCAA Division I men’s basketball. Even with the existence of overt economic and performance-based metrics, the status of a team in NCAA is known to have consequential effects on the behaviors and outcomes of the team [20–22]. Widely agreed-upon status hierarchies also exist for teams and conferences [15, 20]. The field audiences indeed recognize the status inconsistency between a team and its belonging conference as noted: “Gonzaga is a high-major school trapped in a mid-major conference” [23]. Moreover, scheduling games with non-Division I teams provides an opportunity to see how the tendency to form a status-threatening tie is influenced by nested status inconsistency. Our empirical results show that status-inconsistent teams are less likely to schedule games with non-Division I teams than status-consistent teams are. We find that this effect is strongest for teams that belong to the Mid-major conferences. Furthermore, teams that showed high performance in the previous season are less subjective to the negative effect of status inconsistency than others.

Our study contributes to the status literature by theorizing the differential impact of a nested form of status inconsistency on organizational behaviors compared to other forms of status inconsistency. We propose that when an organization’s status differs from that of its belonging group, this nested status inconsistency imposes relational constraints that restrict the organization from resolving the inconsistency. Consequently, the organization experiences status anxiety, leading to adherence to existing norms and avoidance of counternormative behaviors, such as forming status-threatening ties. Moreover, our study provides an additional perspective on the relationship between status and norm conformity. Studies on middle-status conformity, have examined the mechanisms of how middle-status position is often linked to conformity [16, 17]. Our study suggests that the nested form of status inconsistency can be another source of norm conformity. Finally, the study underscores the practical importance of a holistic approach to counter-normative behavior in organizations and athletic teams, emphasizing the need to balance status consistency with other benefits like financial returns and strategic growth opportunities.

## Theory and hypothesis development

### Organizational status and status inconsistency

The status of an organization influences how others perceive and act toward it, subsequently dictating the opportunities and limitations the organization faces [2, 3, 5, 24]. Organizations that engage in multiple status hierarchies then face different sets of opportunities, constraints, and behavioral consequences. When organizations occupy inconsistent status positions across different hierarchies, they are likely to encounter conflicting expectations [8, 9, 11, 12]. The effects of status inconsistency were initially discussed in sociology and psychology literature, focusing on how individuals are affected by status inconsistency and how they respond to it [9, 10]. Recent management studies have begun to explore the effects of status inconsistency on organizations, defined as “the extent to which [organizations] occupy unequally ranked status positions accorded different amounts of prestige in different social systems” [8, p.1023, 11, 12, 25]. These studies examine the unique characteristics of status inconsistency that significantly impact organizational performance and how organizations respond to such inconsistency.

Research in this field generally suggests that status inconsistency has a negative effect on social actors [8, 9, 25]. Early work in psychology and sociology argued that individuals experiencing status inconsistency are more likely to experience psychological stress compared to others [9, 10]. Organizations facing status inconsistency also encounter negative performance due to status ambiguity perceived by external audiences [8, 11, 25]. Given the adverse effects of status inconsistency, previous studies emphasize that social actors often make discretionary efforts to reduce status inconsistency by improving their lagging status position [12] or by withdrawing from participation in the lagging status system [13]. For instance, critically acclaimed actors or actresses in the U.S. film industry are more likely to participate in movies with commercial potential to enhance their position in the commercial status hierarchy [12]. Additionally, social actors attempt to reduce status inconsistency by partially altering the status system [9, 26]. Individuals experiencing status inconsistency were more inclined to vote for the Democratic Party, anticipating a change in the status quo [9]. Our study extends this line of research by highlighting another form of status inconsistency—the nested nature of certain status hierarchies—and examines why and how this form is likely to constrain organizational attempts to resolve status inconsistency.

### Nested form of status inconsistency (SI)

As previously mentioned, we define nested form of status inconsistency as the difference between the status of an organization and the status of the group to which the organization belongs in the same social system. Based on this definition, we first elaborate on the distinction between the nested form of status inconsistency and status inconsistency stemming from different dimensions of status evaluation or audience heterogeneity [8]. Status inconsistency can arise due to variations in different dimensions of status evaluation [9, 10, 25]. An individual may hold unequally ranked positions in terms of income, ethnicity, education, and occupation [9]. Similarly, a bottle of wine can possess different status positions based on various dimensions, such as wine quality rating or geographical origin [25]. Social actors may also encounter status inconsistency when evaluated by different groups of audiences [12, 27, 28]. In the U.S. feature-film industry, professionals often receive disparate evaluations from two distinct audience groups—peer professionals and movie critics [27]. PGA golfers also experience varying status positions depending on whether the audiences are their peers or the general public [28].

In contrast, the nested form of status inconsistency arises from the difference in status between an organization and its belonging group [8, 13]. For instance, a conglomerate may also face status inconsistency if its business units occupy unequal status positions across different markets. Importantly, we assume that the status of a group is composed of the status positions of its individual members [12, 29–31]. Social actors often belong to a group, and their status is largely influenced by this group membership. Based on this notion, past literature has demonstrated the influence of the nested structure of status hierarchies on an actor’s behavior [e.g., 16, 32, 33]. For example, organizational failure or stigmatization strongly influences group members, suggesting that organizational status can serve as either a buffer or a liability for individual members [33]. Moreover, organizational status and individual status can interact and influence actors’ core behaviors, such as security analysts’ firm rating behavior or conductors’ programming choices for an orchestra [32, 34].

Specifically, our focus is on the nested form of status inconsistency from the viewpoint of a subunit organization belonging to the multiunit group. In the context of our study, we explore the nested status inconsistency from the perspective of a team, which serves as the subunit within the multiunit organization known as a conference. Recent studies on status inconsistency have highlighted how inconsistency among organizations within a group can have distinctive effects on organizational behavior and outcomes [8, 13]. While most research on status inconsistency has concentrated on the status of the organization itself, the presence of nested status systems implies that, from the perspective of the subunit organization, its own status is also influenced by the status positions of other sibling organizations within the same group. For instance, a department with higher status than other departments in a business school is likely to face a nested form of status inconsistency, as the status of other departments collectively determines the overall status of the business school to which the department belongs. Similarly, a business unit may face a nested form of status inconsistency when it is regarded differently in the market compared to other business units within the firm. Consequently, such status interdependence between organizations within the group can result in distinct behavioral outcomes for the focal organization.

Importantly, the nested form of status inconsistency serves as a relational constraint on the subunit organization. Due to limited control over the status positions of other sibling organizations within the same group, the focal (subunit) organization faces greater difficulty in reducing the status inconsistency between itself and its belonging group compared to other forms of status inconsistency. Prior research has highlighted various strategies employed by status-inconsistent organizations, such as enhancing lagging status dimensions, divesting status-lagging subunits, or attempting to change the status system [9, 12, 13, 26]. These strategies assume that organizations can effectively reduce status inconsistency through their own actions. However, when the organization’s status inconsistency involves the status positions of other organizations, it becomes more constrained in resolving the status inconsistency through independent actions. Additionally, as audiences often focus on the status of the group to which the organization belongs [35, 36], the organization is particularly susceptible to this constraint imposed by its belonging group, as its individual status alone may not sufficiently influence audience perceptions. Consequently, the status positions of sibling organizations can significantly influence the behavior of the focal organization, leading to status-conscious behavior. We will delve into this topic further in the following section.

### Nested form SI and avoidance of status-threatening ties

Given its nature as a relational constraint, we argue that the nested form of status inconsistency leads to status anxiety and avoidance of status-threatening activities. Status anxiety refers to concerns about being unable to occupying status positions that the focal organization believes to deserve, that is, “a worry that [the organization is] currently occupying too modest a rung or about to fall to a lower one” [13, 17, 18]. Status-anxious organizations become more mindful of their actions and their potential impact on the external audience’s status evaluation [2, 5]. Therefore, organizations facing nested status inconsistency may experience heightened status anxiety and actively avoid engaging in activities that could negatively affect their status positions. Forming a tie with a visible and active low-status actor can pose a significant threat to the status of the focal actor [5]. Organizations generally prefer not to form ties with lower-status partners since the status of a social actor is partially defined by the status of its affiliates [1, 5]. Forming ties with low-status actors is likely to be perceived as a violation of identity codes, leading to heightened scrutiny and a betrayal of commitment to key audiences in the field [37]. These ties nevertheless exist to some extent because of the other benefits that may offset such status-related concerns [38].

We focus on examining the formation of status-threatening ties, which negatively influence the organization’s status, and how the nested form of status inconsistency affects the avoidance of such activities. When processing information, audiences often initially direct their attention to group-level information due to the use of categorical thinking [35, 39]. Fiske and Neuberg [35] propose that people prioritize category-based components, such as social groups, as they have limited cognitive capacity and time when forming impressions. As a result, individuals tend to rely on categorical thinking to facilitate efficient information processing [39]. Similarly, when evaluating the status of an organization, audiences are likely to give precedence to the status of the group to which the organization belongs [33, 34]. As discussed above, group status is the overall status of the group to which the focal organization belongs, which is also determined by status of other organizations within the group. Goode [36, p. 101] notes that:

[P]eople first perceive one another as “members” of such units, their initial behavior and attitudes are determined most by the respect or dispraise they usually give to such social categories or groups.

In this sense, the group status of an organization is often the default reference point for audiences when evaluating the organization [40, 41]. Therefore, when the group membership of each organization in the field is clearly established, the individual status of an organization is likely to be assessed based on its affiliation with the group.

An organization that has a different status from the group is likely to face the risk of status devaluation and experience status anxiety, which in turn leads to the avoidance of status-threatening ties. When an organization deviates from the group mean in terms of status, it is prone to being devalued by others [28]. Such deviation from the group mean hampers the cognitive fluency of the audience when evaluating organizations, resulting in cognitive confusion and ambiguity in the status evaluation process. This overall process can lead to “vertical identity ambiguity,” which is associated with difficulties in making accurate evaluations and receiving negative evaluations from the audience [13]. It is important to note that this process is applicable irrespective of whether the organization’s status is lower or higher than the group’s overall status. When an organization’s status is lower than the group status, it may experience pressure to demonstrate its group membership, which comprises other organizations of higher status. The organization may thus strive to avoid any activities that could draw audiences’ attention and take away its high status derived from group membership. Conversely, when an organization holds a higher status within the group, its mere association with lower-status organizations can negatively impact its own status. This association could be involuntary, yet it still poses a risk to the organization’s status, causing concern about it. Consequently, the organization is likely to endeavor to avoid any behaviors that deviate from the norm and could heighten the likelihood of status devaluation stemming from such associations. In this sense, counternormative behavior is more likely to be conceived as status-threatening when status inconsistency is higher because such actions are likely to be more noticeable when the audience face difficulties in evaluating the focal organization’s status. The risk of devaluation triggers status anxiety as the focal organization feels threatened about its current status position [13, 18]. Consequently, this status anxiety drives the organization to avoid forming ties that may further jeopardize its status, as a means of reducing the risk of being devalued by the audience [18]. Therefore, we propose the following hypothesis regarding the impact of status inconsistency between an organization and its group on the formation of a status-threatening tie:

*Hypothesis 1*: *The greater the difference between the status of an organization and the status of its group*, *the higher the likelihood that the team will avoid forming a status-threatening tie*.

### Nested from SI and moderating factors

#### Status inconsistency effects by group status

We have proposed that the nested form of status inconsistency leads to status anxiety in the focal organization, resulting in the avoidance of status-threatening ties. Now, we posit that the impact of nested status inconsistency is contingent upon the status of the belonging group. As previously discussed, the status of an organization’s belonging group, determined by status of the whole members within the group, serves as the default reference point and influences the evaluation of the organization’s individual status [40, 41]. Thus, the group status operates as an organization’s “membership in sociologically real categories” [42] and can affect its vulnerability to status inconsistency. Consistent with previous studies in the status literature, we categorize group status into high, middle, and low status groups to capture the categorical nature of the status hierarchy [7, 40]. Specifically, we examine how each group membership relates to the extent to which status inconsistency influences engagement in status-threatening activities by influencing the levels of status anxiety.

First, organizations belonging to high-status and low-status groups are less susceptible than those in middle-status groups to the effects of status inconsistency on the avoidance of status-threatening ties, primarily due to the relatively fixed identities of their group membership, which makes them less sensitive to additional status anxiety [7]. High-status organizations, by virtue of their position, are inherently on the top of audiences’ mind [43–45] and their established identity as core members of the social system often serves as a buffer against the status anxiety that could potentially arise from status inconsistency. At the other end of the group status hierarchy, low-status organizations are typically excluded from audiences’ consideration set in the first place [7]. The low-status position inherently makes audiences’ expectations less constraining, thereby making status inconsistency less impactful on their status-threatening activities.

In contrast, organizations in the middle-status groups are likely to be influenced by the negative effects of status inconsistency on the avoidance of status-threatening ties by further increasing the level of status anxiety. The identity of middle-status group is relatively unstable and fuzzy compared to the high- and low-status groups within the field’s status hierarchy, resulting in an inherently uncertain status position [7, 19, 41]. Status inconsistency further increases the risk of devaluation by audiences due to status ambiguity and, in turn, heightens the status anxiety of the organization. Therefore, in our context of NCAA basketball, teams belonging to the middle-status group, known as Mid-major conferences, are more likely than those in the high- and low-status groups to reduce their tendency to engage in status-threatening activities when facing status inconsistency. Accordingly, we hypothesize that:

*Hypothesis 2*: *The effect of status inconsistency on avoidance of status-threatening ties is strongest for organizations in the middle-status group*.

#### Status inconsistency effects by recent high performance

We have examined how the nested form of status inconsistency leads organizations in the middle-status group to avoid status-threatening ties. We further argue that this effect of status inconsistency is contingent upon recent performance. The attention of audiences plays a crucial role in interpreting the actions of organizations and their evaluations in the field [46]. When evaluating organizations, audiences tend to shift their attention to noticeable external signals, such as highly publicized recent performance [47]. We examine how recent high performance affects the negative effect of status inconsistency by shifting the attention of audiences from group-level evaluation to individual-level evaluation. It should be emphasized that performance differs from status in that performance is based on observed merit-based factors, while status relates to one’s social ranking, which is usually accumulated over a long period of time [1, 5, 20]. Importantly, status literature has highlighted that although one’s status may be associated with performance, it is also easy to decouple from merit-based performance [1, 20]. While these two are often difficult to empirically separate, the sports setting allows us to clearly delineate performance from status, as recent objective performance can be separately measured from the prestige accorded to the team [20].

We consider recent high performance as a signal that increases the focal organization’s salience and direct the attention of audiences to the focal organization [47–49], thereby weakening the status anxiety stemming from status inconsistency. Recent high performance makes the organization more prominent within the group, capturing the audiences’ attention [48]. Therefore, heightened salience resulting from recent high performance can shift the focus from group-based evaluation to individual-based evaluation, prompting the audiences to make “dispositional attributions…to account for puzzling inconsistencies” in their evaluation processes [48, p. 137]. In this sense, the positive expectation-violating signal compels the audiences to allocate more attention to resolve the incongruity in their expectations [49, 50]. By emphasizing the individual organization, the cognitive confusion caused by the status difference between the focal organization and its belonging group is likely to be reduced. As a result, status-inconsistent organizations experience less anxiety about forming status-threatening ties when they demonstrate high performance. If a status-inconsistent organization has recently shown high performance, it needs to less worry about the appropriateness of its other activities and have relatively more freedom to engage in status-threatening activities than others [2, 5]. As we argue that the effect of status inconsistency is strongest in the middle-status group, we also propose that this moderating effect of recent high performance is most pronounced for organizations in the middle-status group. Specifically, we hypothesize that recent high performance, such as a high winning percentage in the recent year, weakens the negative effect of status inconsistency for teams in the Mid-major conferences.

*Hypothesis 3*: *Recent high performance weakens the effect of status inconsistency on avoidance of status-threatening ties for those in the middle-status group*.

## Data and methods

### Context: NCAA Men’s basketball

The empirical setting of our study is NCAA Division I men’s basketball from 2000 to 2019. College basketball, which has a history of over one hundred years, has garnered significant public interest, attracting millions of viewers and generating substantial revenues [21]. Two characteristics of the setting are important to note for the purpose of this study. First, the status of a team and/or a conference has a meaningful influence on teams’ outcomes and behaviors in NCAA [20–22]. The status of a team, for instance, often influences the likelihood of receiving an invitation to the NCAA tournament, even when controlling for its performance in a given season [20]. Similarly, the status of a conference, comprising of nine to fourteen teams, is known to have significant effects [21, 51]. High-major conferences, also known as “Power Six” [15], often enjoy privileges, such as positive evaluations in the team selection process for the NCAA tournament, compared to lower-status conferences labeled as “low- or mid-majors” [51, 52]. As we theorized earlier, the audiences in this field typically prioritize the evaluation of a team based on its conference. For instance, Katz [15] notes, “Gonzaga is the only school to move up to high-major status without jumping to a high-level conference. But that won’t stop the masses from still calling them a mid-major,” indicating the primacy of conference status in the audiences’ evaluation of the team.

Second, games with non-Division 1 teams provide an opportunity to explore how status inconsistency affects counternormative behaviors. In addition to games within a conference, each team schedules and plays games with teams from other conferences, known as nonconference games. These nonconference games typically make up approximately one-third of the regular season. Nonconference scheduling is a crucial and complex procedure for teams to improve their performance and gain recognition from interested audiences, including the NCAA committee, journalists, and fans [53]. This paper focuses on games with non-Division I teams that are part of these nonconference games. Despite being considered a negative signal to interested audiences, scheduling games with lower-division teams is common. Washington and Zajac [20, p. 289] find that “playing even the small permitted number of games against these lower-division teams erodes status through negative association.” Fans may also perceive it negatively, as they expect to see Division I basketball in their season tickets and may be disappointed when Division II teams are on the schedule [54]. However, these games are often scheduled due to other benefits such as economic opportunities and the likelihood of easy wins. These benefits are explicitly acknowledged in the field as below:

For schools that don’t want to go on the road or need a specific number of home games *for revenue purposes*, the choice is often between low-level Division I opponents and non-Division I schools

[54, italics added].

Considering the trade-off between status and other benefits, some teams choose to include non-Division I games in their nonconference schedule [55]. Therefore, NCAA Division I men’s basketball provides an appropriate setting to test our hypothesis regarding the effects of status inconsistency (i.e., the difference in status between a team and its conference) on status-threatening behaviors (i.e., scheduling non-Division I games).

### Data, sample and variables

We collected data on NCAA Division I men’s basketball teams from the 2000–2001 season to the 2018–2019 season. The data was obtained from various sources, including sportsreference.com, NCAA.com, OPE Equity in Athletics Disclosure Website database, ESPN.com, CBSsports.com, kenpom.com, USA Today.com, and ESPN College Basketball Encyclopedia. To ensure the consistency of the data, we excluded teams without conference membership in a given season. Additionally, we included one-year lagged variables in our analysis. The final sample consists of 6,006 team-season observations from 353 schools belonging to 34 conferences.

#### Measurements

*Dependent variable*. The dependent variable (*Non-Division I Games*) represents the number of games a team scheduled against non-Division I teams in a given season. These non-Division I games serve as a status-threatening ties because playing against non-Division I teams is perceived as having a negative association with status by the interested audiences.

*Explanatory variables*. To operationalize the status inconsistency variables, we utilized the cumulative ranking points from the final results of the AP Poll to measure the status position of a team, following previous studies [20, 43]. The AP Poll, compiled by the Associated Press since 1948, is based on the ballots of 65 journalists in the field and is considered a reliable indicator of a team’s perceived competency and recognition, reflecting their status in the field [43]. The ranking ranges from 1st to 25th, and we reverse-scored each rank (e.g., 26 for 1st, 1 for 25th, and 0 for unranked). We used the cumulative score from 1985 onward, which is considered the starting point of the “modern era” in this context [56]. For measuring the status of a conference, we calculated the average status scores of the teams, aligning with the definition of group status in our theoretical arguments [7, 57]. To reflect the relative nature of status and ensure comparability between team and conference status, we standardized the measures [16]. To measure status inconsistency, we computed the absolute difference between Team Status and Conference Status, represented as |Team Status—Conference Status|.

To identify whether a team belongs to the middle-status group, we created a dummy variable called *Mid Major*. In NCAA, conferences are often categorized into groups that are widely recognized by the audiences in the field, such as “Power Six,” “Mid Major,” and “Low Major” [15, 51]. Mid-major conferences are perceived as conferences that strive for success but are not part of the Power Six conferences [15, 23, 58]. We categorized the following conferences as Mid-major: American Athletic Conference, Atlantic 10 Conference, Conference USA, Mountain West Conference, and West Coast Conference [59]. These conferences are non-Power Six conferences that have produced teams ranked in the AP Poll to some extent. To capture recent high performance, we used *Winning Percentage* [20]. We opted for winning percentage instead of Rating Percentage Index (RPI) [51] as it is a more intuitive measure that can facilitate the process of shifting audience attention [47, 48]. However, using RPI as the performance variable yielded similar results to our main models. Note that this operationalization of recent performance as observed merit-based factors differs from status, which is rooted in long-term social ranking as mentioned above.

*Control variables*. We included several control variables to address alternative explanations. Firstly, we controlled for team status and conference status, in line with previous research on status inconsistency [8, 12, 26]. This was done to eliminate spurious effects arising from individual team status and conference status. However, the continuous status variables (*Team Status* and *Conference Status*) exhibited high collinearity with the status inconsistency variables, which are linear combinations of the former status variables [12, 26]. To address this issue, we introduced a dummy variable indicating whether a team’s status was above the top 20th percentile in a given season (*High-Status Team*). Similarly, we included the variable *High-status Conference* to account for the independent high-status effect of the conference.

At the team-season level, we included controls for factors that may impact a team’s scheduling choices. Firstly, we controlled for whether the team received an invitation to the NCAA Tournament in the previous year (NCAAT Invitation). The NCAA Tournament, also known as “March Madness,” invites 64 to 68 teams to participate, and receiving an invitation is highly valued by teams and audiences alike as it signifies success and garners public attention [59, 60]. Additionally, we included a variable indicating whether the team received an invitation to the National Invitation Tournament (NIT Invitation), which is a second-tier tournament for teams that did not receive NCAA Tournament invitations. Furthermore, we controlled for the team’s attendance ratio, which is the average home attendance divided by the stadium capacity. Attendance ratio may influence the decision to schedule non-Division I games, as teams with strong fan support may view certain nonconference games as revenue sources [54, 55].

We also considered the influence of the team’s current season schedule on the scheduling of non-Division I games. The overall characteristics of the schedule, including the number of games and the difficulty of the schedule, are likely to impact the team’s decision regarding non-Division I game scheduling [53]. First, we controlled for the total number of regular games (*Total Games*) and the number of non-conference games (*Non-conference Games*) in a season. The number of games reflects the team’s scheduling capacity and may affect their propensity to form ties with unconventional opponents [61]. Second, we included the strength of schedule for nonconference games (*Nonconference SOS*), as the difficulty of games against other teams can influence the decision regarding non-Division I game scheduling. For example, a team that schedules nonconference games against strong opponents may fill the remaining slots with weaker non-Division I teams [54]. The nonconference strength of schedule measure by Ken Pomeroy, commonly used in NCAA basketball research, was used for this variable [62]. We also controlled for whether the team played against Chaminade, a non-Division I team, as part of their regular non-conference schedule (*Played Chaminade*). Chaminade serves as the host of the Maui Invitational, a tournament where many high-status teams compete. In this case, games against Chaminade are less likely to be perceived as status-threatening ties by the audiences.

We also included team-level variables that may influence the team’s decisions. First, we included a dummy variable, *New Team*, which indicates whether the team newly entered Division I during the research period. This variable captures the influence of teams that have recently transitioned to Division I status. Additionally, we included a variable indicating whether the team’s school is public or private (*Public School*). The type of school may impact the team’s financial support from alumni or the public, which could influence their decisions [20]. Furthermore, we considered the impact of conference membership changes on the team’s decision-making. A dummy variable, *Conference Change*, was included to indicate whether the team changed conferences before the start of a season. Changes in conference membership can affect the perception of the interested audiences, potentially influencing the team’s response to status inconsistency [63].

### Statistical analysis

Our dependent variable, *Non-Division I Games*, is a count variable. When dealing with count variables, the assumptions of ordinary least squares (OLS) estimation are violated, and alternative methods such as the Poisson or negative binomial model are more appropriate. The Poisson model assumes that the data does not exhibit overdispersion, meaning that the variance-to-mean ratio is relatively constant across groups [64]. Based on the descriptive statistics presented in Table 1, our dependent variable does not show evidence of overdispersion (mean = 1.06, s.d. = 1.12). Therefore, we applied random-effect Poisson models for the statistical analysis of our hypotheses, employing the XTPOISSON command in Stata 16. To cluster the standard errors at the team level, the vce(robust) option was used. To address the issue of reverse causality and account for the idea that non-Division I games are typically scheduled last [53] before the start of a new season, most variables, except for the current-season variables related to scheduling, are lagged by one season. Additionally, all the models include year-fixed effects to control for any time-specific factors that could influence the dependent variable.

**Table 1:** Descriptive statistics and correlations.

Variables	Mean	S.D.	(1)	(2)	(3)	(4)	(5)	(6)	(7)	(8)	(9)	(10)	(11)	(12)	(13)	(14)
(1) |Team Status—Conference Status|	0.53	0.69														
(2) Mid Major	0.14	0.35	-0.04													
(3) Winning Percentage	0.51	0.17	0.24	0.07												
(4) High-status Conference	0.21	0.41	0.70	-0.20	0.25											
(5) High-status Team	0.21	0.41	0.54	0.02	0.34	0.71										
(6) NCAAT Invitation	0.20	0.40	0.30	0.02	0.60	0.35	0.41									
(7) NIT Invitation	0.10	0.30	0.11	0.06	0.25	0.14	0.13	-0.17								
(8) Attendance ratio	0.54	0.26	0.38	0.13	0.47	0.41	0.48	0.40	0.17							
(9) Total Games	29.42	1.46	0.10	0.05	0.13	0.15	0.15	0.11	0.01	0.06						
(10) Non-conference Games	12.51	2.08	0.04	0.12	0.06	0.02	0.07	0.05	0.01	0.02	0.53					
(11) Played Chaminade	0.01	0.09	0.10	0.01	0.06	0.11	0.14	0.08	0.04	0.09	0.04	0.03				
(12) Non-conference SOS	-0.31	4.94	-0.11	-0.05	0.12	-0.17	-0.07	0.11	0.04	-0.01	-0.01	-0.08	0.01			
(13) New Team	0.05	0.23	-0.10	-0.10	-0.08	-0.12	-0.12	-0.08	-0.05	-0.12	0.04	0.09	-0.02	0.04		
(14) Public School	0.65	0.48	0.08	-0.14	0.02	0.10	0.05	0.04	0.01	-0.08	0.07	-0.03	0.02	0.09	0.05	
(15) Conference Change	0.03	0.17	-0.03	0.00	0.03	-0.04	-0.03	-0.01	0.00	-0.01	-0.01	-0.02	-0.02	-0.01	0.05	0.00

N = 6,006. Coefficients greater or equal to |.03| are significant at p < .05

## Results

Table 1 presents the descriptive statistics and correlations of the variables. The correlations between *|Team Status–Conference Status|* and *High-Status Conference* and between *High-Status Team* and *High-Status Conference* could raise a multicollinearity issue. We calculated the variance inflation factors (VIFs) for all the models. The mean VIFs were below the conventional threshold of ten, indicating that multicollinearity is not likely to be a significant concern in our statistical analyses. To ascertain the robustness of our findings, we additionally estimated the models with orthogonalized status inconsistency and status variables (results available upon request), following the approach used in previous studies on status inconsistency [8, 13]. The results will be available upon request.

We also test Hypotheses 2 and 3 by splitting the sample based on the group status. Table 2 shows the results from the analyses. The results reported in Model 5 are for the subsample that consists only the teams in the Mid-Major conferences. Model 6 and Model 7 show the results for the subsample of only the teams of Power Six and Low Major. The negative effect of status inconsistency is only statistically significant for the subsample of the Mid-major teams in Model.

**Table 2:** Poisson regression models on the number of non-Division I games.

	Model 5	Model 6	Model 7	Model 8	Model 9	Model 10
	Middle	High	Low	Middle	High	Low
|Team Status-Conference Status|	-0.643*	-0.133	-0.540	-1.839***	-0.352	-0.533
	(0.321)	(0.140)	(0.423)	(0.361)	(0.422)	(0.773)
Winning Percentage	0.073	-0.324	0.396***	-0.544	-0.822	0.399
	(0.463)	(0.668)	(0.120)	(0.429)	(1.017)	(0.279)
|Team Status-Conference Status|X Winning Percentage				2.086***	0.326	-0.014
				(0.444)	(0.557)	(1.187)
High-Status Conference	0.036	0.127		0.129	0.124	
	(1.127)	(0.224)		(1.050)	(0.226)	
High-Status Team	-0.359	-0.579	-0.027	-0.389	-0.607	-0.026
	(0.222)	(0.671)	(0.245)	(0.248)	(0.696)	(0.250)
NCAAT Invitation	-0.149	0.024	-0.159*	-0.269†	0.040	-0.159*
	(0.128)	(0.344)	(0.063)	(0.146)	(0.336)	(0.063)
NIT Invitation	-0.347**	0.019	-0.098	-0.390***	0.021	-0.098
	(0.116)	(0.164)	(0.078)	(0.114)	(0.166)	(0.078)
Attendance Ratio	-0.743	-0.879	-0.260	-0.764	-0.901	-0.260
	(1.440)	(1.114)	(0.238)	(1.443)	(1.135)	(0.238)
Total Games	-0.057	0.121	-0.076***	-0.050	0.126	-0.076***
	(0.141)	(0.165)	(0.015)	(0.144)	(0.163)	(0.015)
Non-conference Games	0.115	0.142	0.114***	0.113	0.138	0.114***
	(0.141)	(0.128)	(0.011)	(0.142)	(0.127)	(0.011)
Played Chaminade	1.303***	1.815***	0.307	1.300***	1.817***	0.307
	(0.234)	(0.136)	(0.219)	(0.241)	(0.132)	(0.219)
Non-conference SOS	-0.022*	0.012	0.010	-0.022*	0.011	0.010
	(0.009)	(0.052)	(0.006)	(0.009)	(0.054)	(0.006)
New Team	0.509*	0.212	0.439***	0.530**	0.211	0.439***
	(0.204)	(0.248)	(0.101)	(0.186)	(0.250)	(0.101)
Public School	-0.025	0.439	0.004	-0.045	0.449	0.004
	(0.242)	(0.557)	(0.062)	(0.247)	(0.550)	(0.062)
Conference Change			0.321**			0.321**
			(0.103)			(0.103)
Constant	0.406	-4.993†	0.896*	0.638	-4.718	0.895*
	(2.628)	(2.901)	(0.400)	(2.751)	(3.037)	(0.437)
Year fixed effects	Yes	Yes	Yes	Yes	Yes	Yes
Observations	855	1,299	3,852	855	1,299	3,852
Number of schools	69	78	246	69	78	246
Log pseudolikelihood	-832.0	-615.9	-4930	-827.3	-615.7	-4930
Wald χ2	394.1	465.6	983.1	492.2	567.4	1006

*** p<0.001,

** p<0.01,

* p<0.05,

^†^ p<0.10 (two tailed, robust standard errors in parentheses)

**Fig 1:**
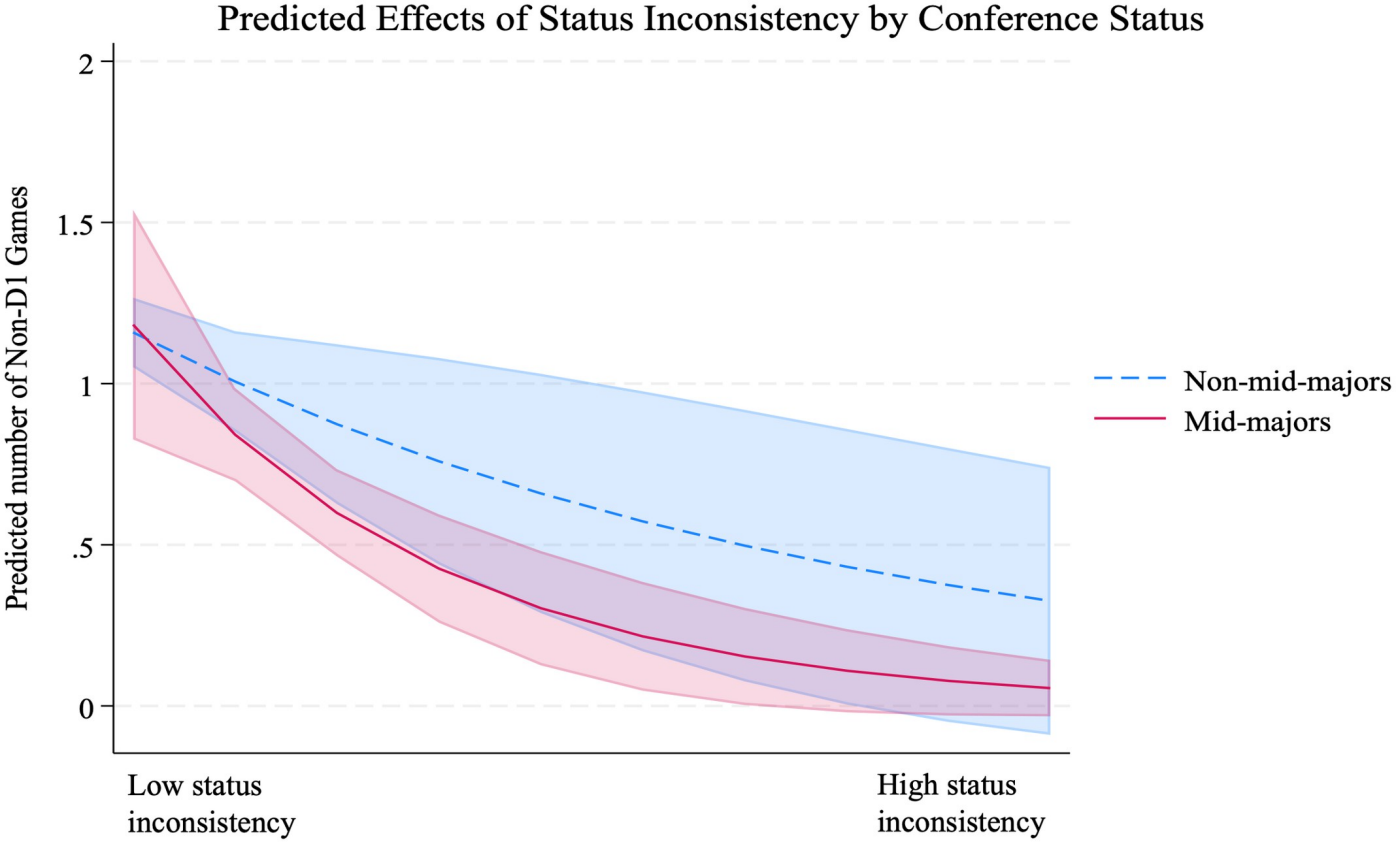
The effect of status inconsistency for Mid Major teams (Model 3).

Table 3 presents the results of the Poisson panel regression analyses on the number of non-Division I games. Model 1 includes the control variables only, and there are some noteworthy results. The high-status variables at the team and conference levels show a negative association with the number of non-Division I games. Additionally, an invitation from the NCAA Tournament in the previous season has a negative and significant effect. These findings indicate the counter-normative aspect of scheduling non-Division I games. Model 2 includes the status inconsistency variable to test Hypothesis 1, which predicted the negative effects of status inconsistency on the number of non-Division I games. The results show that status inconsistency has a negative and significant effect on the number of non-Division I games scheduled (-0.405; *p* = .025), providing support for Hypothesis 1. According to Model 2, a one-standard deviation increase in status inconsistency for a team is associated with a 24 percent decrease in the number of non-Division I games (exp[-0.405*0.69] = 0.76). Hypothesis 2 predicted that the effect of status inconsistency is strongest for the organizations in the middle-status group. Model 3 shows that being a member of the Mid-major conferences weakens the negative effect of status inconsistency (-0.398; *p* = .025), providing strong support for Hypothesis 2. Fig 1 illustrates that teams belonging to Mid-major conferences exhibit a lower tendency compared to teams not belonging to Mid-major conferences. Hypothesis 3 predicted that the high performance in the previous season weakens the negative effect of status inconsistency on the tendency to form status-threatening ties, especially for those in mid-major conferences. As shown in Model 4, the three-way interaction for a team in the middle-status group is significantly positive (2.212; *p* < .001), supporting Hypothesis 3.

**Table 3:** Poisson regression models on the number of non-Division I games.

	Model 1	Model 2	Model 3	Model 4
|Team Status-Conference Status|		-0.405*	-0.282†	-0.237
		(0.181)	(0.146)	(0.201)
Mid Major		-0.104	0.021	0.729***
		(0.164)	(0.168)	(0.213)
Winning Percentage		0.388***	0.386***	0.478***
		(0.117)	(0.117)	(0.127)
|Team Status-Conference Status| X Mid Major			-0.398*	-1.657***
			(0.177)	(0.357)
|Team Status-Conference Status| X Winning Percentage				-0.081
				(0.340)
Mid Major X Winning Percentage				-1.367***
				(0.291)
|Team Status-Conference Status| X Mid Major X Winning Percentage				2.212***
				(0.500)
High-Status Conference	-0.976***	-0.654*	-0.796**	-0.802**
	(0.204)	(0.274)	(0.267)	(0.260)
High-Status Team	-0.707**	-0.623*	-0.617***	-0.615**
	(0.254)	(0.247)	(0.184)	(0.202)
NCAAT Invitation	-0.092*	-0.164**	-0.162**	-0.167**
	(0.046)	(0.063)	(0.060)	(0.062)
NIT Invitation	-0.086	-0.149*	-0.144*	-0.140*
	(0.059)	(0.068)	(0.066)	(0.064)
Attendance Ratio	-0.341	-0.384†	-0.396†	-0.390†
	(0.228)	(0.217)	(0.214)	(0.218)
Total Games	-0.064***	-0.067***	-0.068***	-0.067***
	(0.014)	(0.014)	(0.014)	(0.014)
Non-conference Games	0.115***	0.115***	0.115***	0.115***
	(0.010)	(0.009)	(0.009)	(0.009)
Played Chaminade	1.237***	1.318***	1.340***	1.333***
	(0.179)	(0.174)	(0.176)	(0.177)
Non-conference SOS	0.010*	0.008†	0.008†	0.008†
	(0.005)	(0.004)	(0.004)	(0.004)
New Team	0.422**	0.383**	0.384**	0.384**
	(0.157)	(0.147)	(0.146)	(0.147)
Public School	0.391***	0.390***	0.398***	0.405***
	(0.083)	(0.086)	(0.086)	(0.085)
Conference Change	0.029	0.005	0.007	0.007
	(0.059)	(0.059)	(0.059)	(0.059)
Constant	0.623	0.680†	0.686†	0.616
	(0.446)	(0.405)	(0.402)	(0.418)
Year fixed effects	Yes	Yes	Yes	Yes
Observations	6,006	6,006	6,006	6,006
Number of schools	353	353	353	353
Log pseudolikelihood	-6468	-6444	-6441	-6433
Wald χ2	898.8	975.7	997.8	1055

*** p<0.001,

** p<0.01,

* p<0.05,

^†^ p<0.10 (two tailed, robust standard errors in parentheses)

**Fig 2:**
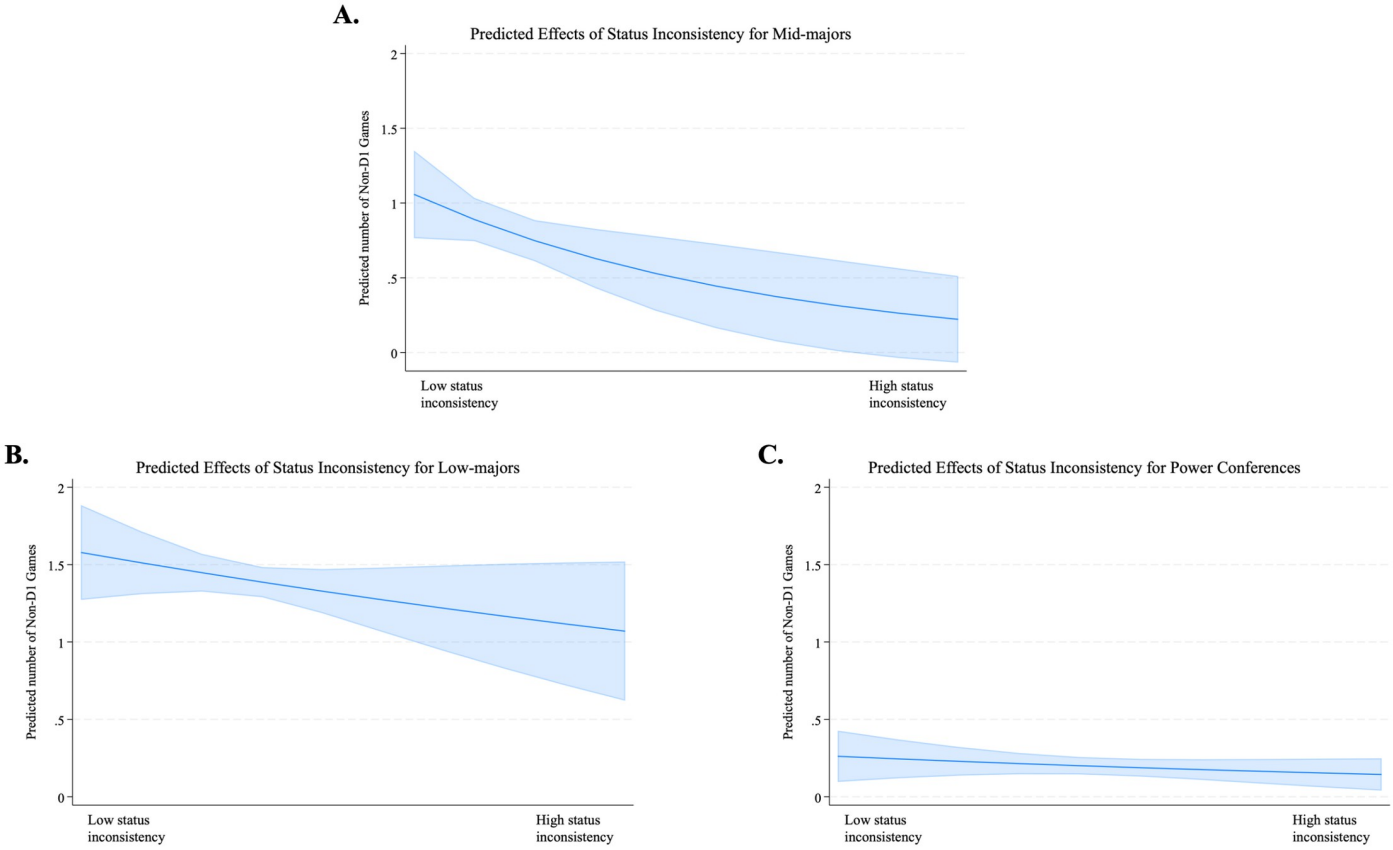
The effects of status inconsistency by group status.

**Fig 3:**
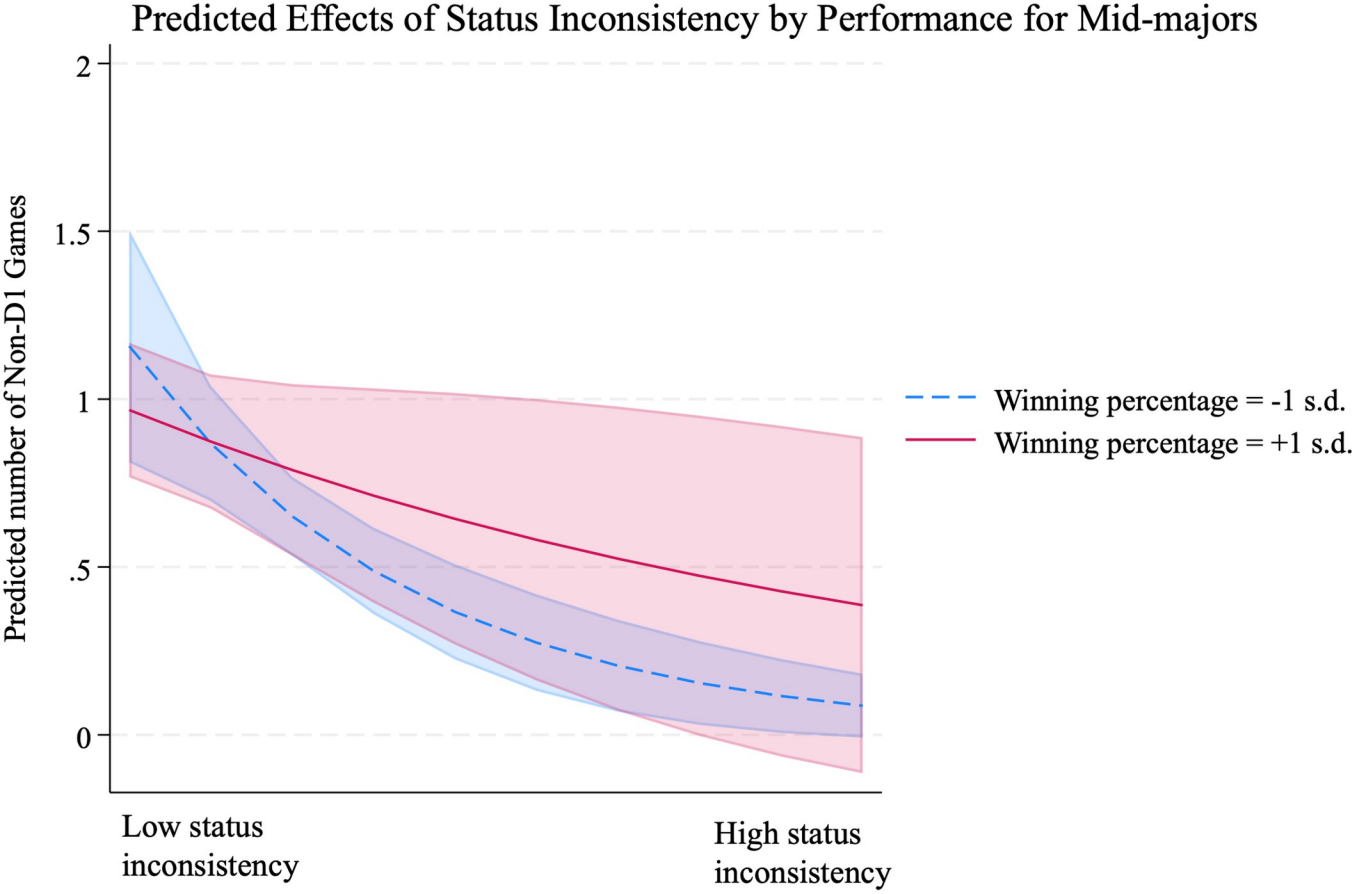
The moderating role of high performance (Model 8).

5 (-.643; p = .045), providing additional support for Hypothesis 2. The effect of status inconsistency was not significant for the subsample of Power Six teams in Model 6 (-.132; p = .345) and for the subsample of Low Major teams in in Model 7 (-.540; p = .201). Fig 2 shows the graph based on Model 5,6, and 7. These results indicate that while higher conference status constrains scheduling non-Division I games overall in the first place (also corroborated with negative main effect of *High-conference Status* in all models), the overall low (high) number of non-Division I games for high-(low-)status conferences does not differ significantly across different levels of status inconsistency. This also implies that status inconsistency has limited influence for both high-status groups, due to their strong core identity commitment, and low-status groups, given their lesser core identity concerns. Meanwhile, the figure demonstrates that the effect of status inconsistency is not only significant for mid-major teams, but it is also the strongest, displaying the steepest slope of difference. The effect of |Team Status—Conference Status| X Winning Percentage in Model 8, which includes only the teams from mid-major conferences, also exhibits a significant positive effect (2.086; p < 0.000). However, the effects of |Team Status—Conference Status| X Winning Percentage in the samples of high-status and low-status conferences (Model 9 and Model 10) are not statistically significant (0.326; p = 0.558 and -0.014; p = 0.991, respectively), providing additional support for Hypothesis 3. Fig 3 illustrates that teams with a winning percentage one standard deviation above the mean are significantly less influenced by the negative effect of status inconsistency within the Mid-major conferences.

## Discussion and conclusion

In this study, we explored how the nested form of status inconsistency influences organizations’ inclination to form a status-threatening tie. When an organization’s status is partly defined by the status positions of other sibling organizations in the same group, it may face challenges in addressing the status inconsistency issue. This nested form of status inconsistency is likely to induce status anxiety and drive the focal organization to adhere more strictly to existing norms, such as avoiding additional status-threatening behaviors. We further hypothesized that the negative effect of status inconsistency would be most pronounced for organizations in the middle-status group due to their inherent positional uncertainty. Additionally, we proposed that recent high performance could attenuate the effects of status inconsistency by diverting the audiences’ attention from the inconsistency to the organization’s individual attributes. To investigate these hypotheses, we utilized data from NCAA men’s basketball. Our findings supported nested status inconsistency between a team and its affiliated conference reduces the likelihood of scheduling non-Division I teams, especially for those in the Mid-major conference. We also showed that the impact of status inconsistency was mitigated when teams had high performance in the previous season.

Our study makes several important theoretical and practical contributions. First, it contributes to the organizational status literature by expanding our understanding of nested status positions, where the status of a focal actor’s belonging group exerts influence on the focal actor’s status. While previous research has acknowledged the importance of such nested status positions [14, 30, 31], their specific impact has remained understudied. The few studies that examine the role of nested status positions usually emphasize that the influence of individual status is contingent on group status, showing that it can buffer group members, allowing more leeway for deviant behavior [16, 40, 41]. Conversely, we argue that group status could also operate as a structural constraint when considering the status difference between the focal actor and their belonging group, which can trigger heightened status anxiety. In other words, our study highlights that group status could also be a liability when the focal actor exhibits large status difference between the group.

Our study also sheds light on the research on organizational status inconsistency. While status inconsistency on individual actors, recently organizational scholars have started to examine status inconsistency effects for organizations [8, 13, 25]. The recent line of work exploring behavioral consequences of organizational status inconsistency primarily focus on how organizations actively strive to reduce it due to its negative consequences on organizational performance [13, 26]. This line of work usually focuses on how the status inconsistency among subunits of a higher-level group (e.g., business school) shape its outcomes and actions. In contrast, our study offers a more nuanced understanding of status inconsistency from the perspective of the subunit as the focal organization (e.g., department) based on the nature of nested status positions.

By theorizing a new mechanism that shows a distinctive source of conformity behavior, our study contributes to the research on the relationship between status and conformity. The literature on middle-status conformity examines why and how middle-status actors are most likely to confront the strongest pressures to conform to the existing norms and expectations of how the social actors should behave [7]. Although a few studies examine when and how conformity pressures on middle-status actors can be relaxed [19, 27, 65], conformity pressures, especially on middle-status actors, have been observed in diverse settings, including the law markets [7, 37], and the newspaper industry [19]. Extending this line of research, our study suggests that nested status inconsistency can be another important source of conformity, independent of the middle-status conformity pressures. In unreported analysis, we included the team status variable and its square term into the main model to test the existence of the middle-status conformity pressures and an inverted U-shaped relationship between status and forming a status threatening tie was found in this model. This unexpected result may mirror the findings of Jensen [65], which found that in the Danish movie industry in the late 1960s and early 1970s, middle-status actors and actresses were most likely to participate in the legitimization of an illegitimate movie genre, sex comedy. The tradeoff between legitimacy power and opportunity costs made middle-status ones, that is, those with enough legitimacy power and not-so-high opportunity costs to most actively participated in sex comedy movies. We expect that middle-status teams in our setting are most likely to schedule non- Division I games with the aforementioned tradeoff between status loss and economic benefits of scheduling non- Division I games. Interestingly, even with these team status variables, the effect of status inconsistency variable still remains in the expected direction. Even with the tradeoff, the result suggests that those facing nested status inconsistency are least likely to engage in any non-conforming activities, indicating another important, but unexplored source of conformity pressures.

Our study also has practical implications for organizations that may face situations involving nested forms of status inconsistency, such as business units within firms, departments within schools, and project groups like VC firms within syndicate groups. Our study highlights the need for a more holistic view of counter-normative behavior, involving the trade-off between status and other benefits such as short-term financial returns for organizations facing nested forms of status inconsistency [5]. In sports contexts like our setting, it is suggested that athletic teams need to adopt a holistic approach when considering the implications of their status inconsistency, group status (e.g., conference, league, associations, etc.), and recent performance on scheduling. While structural constraints arising from status inconsistency influence audience perceptions and actions, teams can also leverage their recent performance to adopt strategic scheduling practices that align with their desired outcomes and goals. This flexibility allows teams to carefully consider the trade-offs between maintaining status consistency and capitalizing on opportunities for growth and visibility. These implications can also be applied to other contexts at a broader level, guiding actions that involve both audience concerns and economic benefits—e.g., a business unit’s strategic alliance partner choice when facing status inconsistency within its firm.

In conclusion, our research offers valuable insights into the complex relationships among status inconsistency, group status, recent performance, and counternormative behaviors in the sport industry. By considering these dynamics, organizations facing the nested form of status inconsistency can make informed decisions that optimize their potential for success and positively impact both their athletic and academic programs. This study opens avenues for further research on the broader implications of status inconsistency and its effects on organizational behavior.

## Supporting information

S1 File Relevant data. (XLSX)
